# Conserved Pheromone Production, Response and Degradation by *Streptococcus mutans*

**DOI:** 10.3389/fmicb.2019.02140

**Published:** 2019-09-13

**Authors:** Antonio Pedro Ricomini Filho, Rabia Khan, Heidi Aarø Åmdal, Fernanda C. Petersen

**Affiliations:** ^1^Department of Physiological Science, Piracicaba Dental School, University of Campinas, Piracicaba, Brazil; ^2^Department of Oral Biology, Faculty of Dentistry, University of Oslo, Oslo, Norway

**Keywords:** pheromone, streptococcus, competence, natural transformation, quorum-sensing, CSP, XIP, ComS

## Abstract

*Streptococcus mutans*, a bacterium with high cariogenic potential, coordinates competence for natural transformation and bacteriocin production via the XIP and CSP pheromones. CSP is effective in inducing bacteriocin responses but not competence in chemically defined media (CDM). This is in contrast to XIP, which is a strong inducer of competence in CDM but can also stimulate bacteriocin genes as a late response. Interconnections between the pathways activated by the two pheromones have been characterized in certain detail in *S. mutans* UA159, but it is mostly unknown whether such findings are representative for the species. In this study, we used bioassays based on luciferase reporters for the bacteriocin gene *cipB* and the alternative sigma factor *sigX* to investigate various *S. mutans* isolates for production and response to CSP and XIP pheromones in CDM. Similar to *S. mutans* UA159, endogenous CSP was undetectable in the culture supernatants of all tested strains. During optimization of the bioassay using the *cipB* reporter, we discovered that the activity of exogenous CSP used as a standard was reduced over time during *S. mutans* growth. Using a FRET-CSP reporter peptide, we found that *S. mutans* UA159 was able to degrade CSP, and that such activity was not significantly different in isogenic mutants with deletion of the protease gene *htrA* or the competence genes *sigX*, *oppD*, and *comR*. CSP cleavage was also detected in all the wild type strains, indicating that this is a conserved feature in *S. mutans*. For the XIP pheromone, endogenous production was observed in the supernatants of all 34 tested strains at peak concentrations in culture supernatants that varied between 200 and 26000 nM. Transformation in the presence of exogenous XIP was detected in all but one of the isolates. The efficiency of transformation varied, however, among the different strains, and for those with the highest transformation rates, endogenous XIP peak concentrations in the supernatants were above 2000 nM XIP. We conclude that XIP production and inducing effect on transformation, as well as the ability to degrade CSP, are conserved functions among different *S. mutans* isolates. Understanding the functionality and conservation of pheromone systems in *S. mutans* may lead to novel strategies to prevent or treat unbalances in oral microbiomes that may favor diseases.

## Introduction

Natural genetic transformation is widely distributed in bacteria. In streptococci it occurs during a genetically programmed differentiated state called competence. During this state the bacteria become capable of taking up DNA from the environment and incorporate it into their genomes. The capacity for natural transformation has been reported for more than 80 bacterial species (Johnsborg et al., 2007; Johnston et al., 2014). Among streptococci, competence for natural transformation includes most species of the mitis, salivarius, bovis, anginosus, and mutans groups (Johnsborg et al., 2007; Fontaine et al., 2010; Desai et al., 2012; Morrison et al., 2013). The core of the machinery necessary for streptococcal transformation relies on the transcription of the conserved alternative sigma factor SigX, also known as ComX. SigX orchestrates a core response in streptococcal species characterized by the induction of 27 to 30 genes (Khan et al., 2016). The functions of the core genes are predominantly related to transformation, most of them coding for competence effector proteins for DNA binding, uptake and recombination (Li et al., 2001; Mashburn-Warren et al., 2010; Khan et al., 2016).

*Streptococcus mutans* is a member of the mutans group and part of the human oral microbiota. Environmental factors disturbing the ecological balance in the oral cavity, such as increased sugar intake, may favor *S. mutans* growth. The *S. mutans* acidogenic and aciduric properties may then contribute to tooth demineralization and dental caries (Marsh et al., 2011). Natural transformation was first reported in *S. mutans* in 1981 (Perry and Kuramitsu, 1981). This and later studies showed that transformation in *S. mutans*, at least in the laboratory setting, is restricted to a limited range of strains and depends on environmental conditions not yet fully understood (Perry and Kuramitsu, 1981). Studies investigating the *S. mutans* pan-genome have recently revealed the ubiquity of *sigX* and competence effector genes in *S. mutans* (Cornejo et al., 2013; Palmer et al., 2013; Song et al., 2013), indicating that competence may be a conserved feature in *S. mutans*. Moreover, the extensive horizontal gene transfer observed in the genomes of *S. mutans* clinical isolates (Cornejo et al., 2013) indicates that transformation occurs in their natural habitat and may be a widespread feature in the species.

**FIGURE 1:**
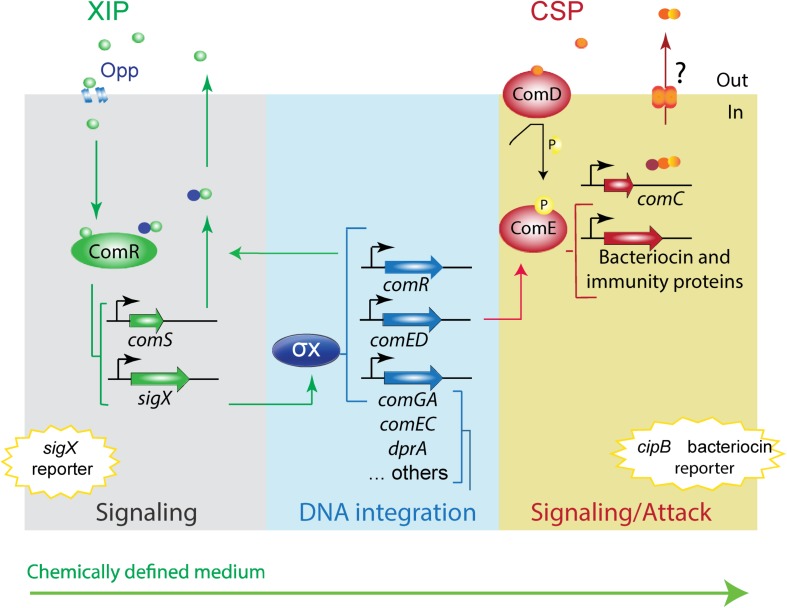
Model of *S. mutans* XIP pheromone signaling pathway in peptide-free chemically defined medium. The model is based on the UA159 *S. mutans* strain. The *S. mutans* XIP pheromone is exported outside of the cells. Upon reaching a certain threshold, XIP is internalized via the Opp permease, and binds to the ComR regulator to activate *comS*, thus creating a positive feedback loop. ComS is the precursor of XIP but can also function in its unprocessed form as an internal signal to activate the ComR-pheromone signaling pathway. The ComR-pheromone complex activates *sigX*. This codes for the alternative sigma factor X (σX). σX is the master regulator of competence for natural transformation that regulates the expression of the streptococcal core set of genes involved in the competence response, most of them with functions that enable DNA acquisition and integration into its genome. σX induces also *comR* (Khan et al., 2017) and *comED* (Reck et al., 2015; Son et al., 2015; Khan et al., 2017). ComED is a two-component system comprising the histidine kinase ComD and the cognate response regulator ComE. ComD autophosphorylates upon CSP pheromone binding and transfers the phosphoryl group to ComE, which in turn induces the expression of bacteriocin and immunity proteins, including the gene for the non-lantibiotic bacteriocin *cipB*. In rich media, *cipB* expression is required for induction of *sigX* by an as yet unknown mechanism. In CDM, CSP induces the ComED pathway, but it is unknown whether the CSP pheromone system is endogenously activated. It is also unknown why in CDM *cipB* is stimulated by CSP, but not the competence response. The conservation of the CSP and XIP signaling pathways in CDM were examined in this study using *sigX* and *cipB* luciferase reporter bioassays.

In *S. mutans* the competent state is triggered by two linear peptides, the CSP (competence stimulating peptide) (Li et al., 2001) and the XIP (*sigX*-inducing peptide) (Figure 1; Mashburn-Warren et al., 2010). CSP was the first described pheromone and, until recently, the only one known to activate the competence system for genetic transformation in *S. mutans*. The *comC* gene, which encodes CSP, is downstream of the *comDE* operon, encoding the ComD histidine kinase and the ComE response regulator. At least in the synthetic form, CSP is thought to bind to the ComD histidine kinase of the ComDE two-component system, leading to phosphorylation of the ComE response regulator (Peterson et al., 2004). Phosphorylated ComE directly up-regulates the transcription of clusters of bacteriocin-related genes (Petersen et al., 2006; Mashburn-Warren et al., 2010; Federle and Morrison, 2012; Khan et al., 2016). These include among others the SMU_1914 gene (also known as *nlmC* and *cipB*), encoding mutacin V, and the putative immunity protein SMU_1913, found in a *comE* downstream region. Mutacin V has been proposed to link the early CSP bacteriocin-inducing response to the competence response (Ween et al., 1999; van der Ploeg, 2005; Kreth et al., 2007; Dufour et al., 2011; Hung et al., 2011) by mechanisms that remain elusive. Essential for competence development is the activation of the XIP pheromone encoding gene, *comS*, followed by the upregulation of the alternative sigma factor SigX, the master core regulator of competence. In rich media, ComS seems to be processed to XIP inside the cells. Intracellular XIP and/or ComS then binds to ComR to activate the competence pathway (Underhill et al., 2018). The predicted CSP is in general conserved among *S. mutans* strains (Li et al., 2001; Petersen et al., 2006), and there are indications that *S. mutans* may form a single CSP pherotype (Petersen et al., 2006; Hossain and Biswas, 2012). The C-terminal of the CSP precursor (ComC) is exported outside of the cells, where it is further processed into the active 18 amino acid peptide (18-CSP) (Petersen et al., 2006; Hossain and Biswas, 2012).

The XIP pheromone system is found in several streptococci (Gardan et al., 2009; Fontaine et al., 2010; Mashburn-Warren et al., 2010; Fleuchot et al., 2011; Morrison et al., 2013). In *S. mutans* UA159 the XIP pheromone encoded by *comS* has been identified in culture supernatants grown in chemically defined medium (CDM) lacking peptides (Mashburn-Warren et al., 2010; Khan et al., 2012). XIP is produced as a pro-peptide (ComS) that is possibly exported and processed into the active XIP (N-GLDWWSL) (Mashburn-Warren et al., 2010; Desai et al., 2012; Khan et al., 2012). The presence of an exporter has, however, never been demonstrated, and recent studies indicate that release of XIP or ComS by autolysis is sufficient to promote intercellular communication in CDM (Kaspar et al., 2017). Under such conditions, response to the XIP depends on the Opp oligopeptide permease system, indicating that the peptide is internalized (Mashburn-Warren et al., 2010). Once inside the cells XIP is thought to bind to the Rgg-like regulator ComR to activate transcription of *sigX* and *comS*. In contrast with XIP, CSP shows no activity or low potency in triggering competence in CDM (Desai et al., 2012; Wenderska et al., 2012; Reck et al., 2015) but can still induce the expression of bacteriocin-related genes of the ComE regulon (Reck et al., 2015). When it comes to transformation, the use of synthetic CSP has led to higher levels of *S. mutans* transformation (Gaustad and Morrison, 1998; Petersen and Scheie, 2010) but has not extended the range of strains transformed in the absence of the synthetic pheromone. As for the synthetic XIP, evidence for competence induction and endogenous pheromone production has so far been restricted to the reference strain UA159, with few exceptions (Palmer et al., 2013).

In this study we investigated the functional conservation of the *S. mutans* CSP and XIP pheromone signaling systems in CDM. Extracellular CSP activity was not detected in any of the tested strains. Exposure to synthetic CSP revealed that *S. mutans* can indeed degrade CSP, a behavior that was conserved in the examined strains, and that at least in strain UA159 it was not abolished by deletion of *sigX*, *comR*, *oppD*, or the *htrA* protease gene. For the XIP pheromone, endogenous production was found in all tested strains, and transformation was found in all but one of the isolates grown in CDM. The results thus indicate the presence of a single *S. mutans* XIP pherogroup and suggest that *S. mutans* has a conserved ability to suppress CSP activity in CDM.

## Materials and Methods

### Bacterial Strains and Media

The *Streptococcus mutans* strains used in this study and their relevant characteristics are listed in Table 1. The strains used in the study were selected from the collection of strains in our laboratory, representing strains known to be transformed (UA159, V403, OMZ175, LML-2, LML-4, GS5, NG8, BM71, LT11) and others in which genetic competence has not yet been characterized. Todd-Hewitt broth (THB; Becton Dickinson) was used to grow all the strains used for DNA extraction and DNA sequencing. Chemically Defined Medium (CDM) (Mashburn-Warren et al., 2010) was used to perform the genetic transformation assays and bioassay to measure the activity of exogenously added CSP in the supernatants of *S. mutans* and also to verify and quantify the concentration of extracellular native XIP in culture supernatants (Desai et al., 2012; Khan et al., 2012). The carbohydrate source was glucose at 1% final concentration, unless specified. The antibiotics erythromycin, kanamycin and spectinomycin were used at final concentrations of 10, 500, and 500 μg mL^–1^, respectively. THB agar and THB agar supplemented with erythromycin were used to enumerate the total and transformed number of *S. mutans*.

**TABLE 1:** Strains and plasmid used in this study.

**Strain/plasmid**	**Relevant characteristics**	**References**
***S. mutans* strains**		
UA159 ^a^	Wild type, transformable strain, Erm^S^	Murchison et al., 1986; Ajdic et al., 2002
LT 11	UA159 derivative, highly transformable	Tao et al., 1993; Petersen et al., 2005
BM71	Caries lesion isolate	Cvitkovitch et al., 1995
Ingbritt	Serotype c	Perch et al., 1974; Perry and Kuramitsu, 1981; Petersen and Scheie, 2000
OMZ 175^a^	Serotype f	Perch et al., 1974; Perry and Kuramitsu, 1981; Petersen and Scheie, 2000; Cornejo et al., 2013
UA130	Serotype c	Murchison et al., 1986; Petersen and Scheie, 2000
V403^ a^	Serotype c; pVA403	Lindler and Macrina, 1986; Petersen and Scheie, 2000; Palmer et al., 2013
NG8	Serotype c	Knox et al., 1986
UAB90 (ATCC 31987)	Serotype c	
NCTC 10449^Ta^	Type strain	Coykendall, 1971; Perch et al., 1974; Song et al., 2013
KM1, 348, 357, 371, 388, 409, 415, 467, 503, 73A2, M1, M4, M6, GW2, GW37	Clinical isolates	Petersen and Scheie, 2000; Petersen et al., 2006
GS-5	serotype c	Perch et al., 1974; Waterhouse and Russell, 2006; Biswas and Biswas, 2012
LML-2, LML-4^a^, LML-5^a^, At10	Clinical isolates	Waterhouse and Russell, 2006; Cornejo et al., 2013
GUV-1	GS-5 derivative, fluoride resistant	van Loveren et al., 1993
CM7	Clinical isolate	
Serogroup C	Clinical isolate	
IB 78	Clinical isolate	
SM004	UA159:Δ*comC*:*kan*; Kan^R^	Petersen et al., 2006
SM059	UA159:Φ(P*_*cipB*_-*luc) Spc^R^	Khan et al., 2012
SM066	UA159:Δ*comR*:*spc*; Spc^R^; DNA source MW02 Mashburn-Warren et al., 2010	This study
SM067	UA159:Δ*oppD*:*spc*, Spc^R^; DNA source MW04 Mashburn-Warren et al., 2010	This study
SM068	UA159: Φ(P*_*sigX*_-*luc) Spc^R^	Khan et al., 2012
SM091	UA159:Δ*comS*:*erm*; Φ(P*_*sigX*_*-luc) Erm^R^, Spc^R^	Khan et al., 2012
SM121	UA159:Δ*sigX*:*kan*; Kan^R^	This study
SM165	UA159: Δ*htrA*:*kan*; Kan^R^	This study
**Plasmid**		
pVA838	Em^R^; streptococcal replicative plasmid	Macrina et al., 1982
**PCR amplicon**		
aRJ02	Δ*dexA*:*kan*	Morrison et al., 2015

*Erm, erythromycin; Spc, spectinomycin; Kan, kanamycin. ^*a*^Genome sequenced strains. ^*T*^, type strain.*

### Construction of Mutants

The deletion mutants were constructed using the PCR-ligation mutagenesis strategy (Lau et al., 2002). Sequence information was obtained from the *S. mutans* UA159 genome. Mutants with deletion of *sigX* and *htrA* were constructed. *Asc*I or *Fse*I sites were incorporated into the 5′-ends of the oligonucleotide primers and both ends of the resistance cassette. The kanamycin resistance cassette was amplified using primer pair FP001-FP068. The *comX* flanking regions were amplified with the primers pairs FP462 (CTTGGTAGCAGGAGAGCAC), FP463 (AAAG CACAGCCTGCTTCAAT) and FP714 (TGCCGAACA CAGCAGTTAAG), FP715 (CATTCCCTCTTGTTGCCAAT). The *htrA* flanking regions were amplified with the primers pairs FP807 (TCCCTCCAATAACGAAGGTCA), FP808 (GGTAAGT GTTGA TATGACCCCT) and FP809 (GAAGGTAGCGTCTA TCAGCGA), FP810 (GCAGTCGAGGTTGATAGGGA). The resultant amplicons were digested with *Asc*I or *Fse*I whereas the kanamycin resistance cassette was digested with both enzymes. The upstream and downstream amplicons of target genes were ligated to the kanamycin cassette using T4 DNA ligase. The two ligated products were mixed and PCR amplified with distal primers. The resultant amplicons were used to transform *S. mutans*. Gene deletion was confirmed by PCR amplification and gel electrophoresis.

### DNA Sequence Analysis

The chromosomal DNA of *S. mutans* strains were isolated as previously described (Petersen and Scheie, 2000). The primers FP678 (5′-ATGCGGAAGCTAAAAAGAGC-3′) and FP679 (5′-TCCAGTCTTCCTATCTGAGCAA-3′) were used to amplify a region of 431 bp, which contains the tRNA transcriptional terminator of *comR* (9 bp stem, a 4 nt loop and a T-rich region at the 3′ side of the stem-loop), the *comS* promoter and the *comS* sequence. First, a PCR was performed (20 μL reaction volume containing 2.5 mM MgCl_2_, 2 pmol of each primer, 0.2 mM of each dNTP, 2 μL of 10× buffer, 50–100 ng of genomic DNA and 2 units of TaqDNA polymerase using a thermocycling profile of initial denaturing period of 3 min at 94°C; followed by 30 cycles of 30 s at 94°C, 30 s at 55°C and 2.5 min at 72°C; with a final extension period of 3 min at 72°C and the products were visualized on a 1% agarose gel to check the amplified fragments by the primers (Petersen and Scheie, 2000). The sequencing was performed bi-directionally in a single experiment for all the samples using BigDye^®^ Terminator v1.1 Cycle Sequencing Kit (Applied Biosystems, Foster City, CA, United States) on an ABI 3730 DNA analyzer (Applied Biosystems, Foster City, CA, United States). All the sequence analysis was done using the Sequencher 5.0 software (Gene Codes, Ann Arbor, MI, United States). The electropherograms were checked and comparative analysis of the bidirectional sequences was performed using the *S. mutans* UA159 sequence as a reference.

### Bioassay of CSP and XIP Activity in Culture Supernatants

The CSP bioassay was performed to measure the production of native CSP and activity of exogenously added CSP in the supernatants of *S. mutans* during growth, and the XIP bioassay was to verify and quantify the concentration of extracellular native XIP in culture supernatants. A P*_*cipB*_*-luc reporter (SM059) was used in the CSP bioassay as previously described (Khan et al., 2012), and a P*_*sigX*_*-luc reporter in a *comS* deletion background (SM091) was used to perform the XIP bioassay as described by Desai et al. (2012), with slight modifications (Khan et al., 2012).

To estimate CSP production and response, the supernatants were collected by 10× dilution of precultures at OD_600_ of 0.6 in CDM containing 10% bovine serum albumin (BSA). When added, 250 nM CSP concentration was used. Supernatants were collected for 6 h during growth. For XIP production and response, overnight cultures of *S. mutans* strains were diluted in fresh CDM to an optical density at 600 nm (OD_600_) of 0.05 and incubated in air at 37°C. At each hour, from the second until the 10 h of bacterial growth, the OD_600_ was measured and 1 mL of the culture was centrifuged (10,000 *g* for 10 min at 4°C) to collect the supernatant. The indicator strains SM059 and SM091 were grown in CDM to an OD_600_ of 0.05 and stored in microcentrifuge tubes with 10% of glycerol at −80°C. The entire assay was performed with stock cultures from the same batch. The bioassay was performed adding 10 μL of each culture supernatant to 50 μL of the indicator strain, 40 μL fresh CDM and 20 μL of 1.0 mM D-luciferin (Synchem, Felsberg-Altenburg, Germany) in a flat-bottom 96-well plate (Nunc, Rochester, NY, United States). Luminescence was measured by reading the plates in a multidetection microplate reader (Synergy HT; BioTek Instruments, Winooski, VT, United States). To quantify the XIP concentrations in the culture supernatants, standard curves were performed at the same time, replacing the culture supernatant with 10 μL of CDM-standard solutions with different XIP concentrations. CDM without synthetic peptides was used to obtain background values that were subtracted from the sample values. For strains exhibiting no XIP activity in the supernatants, the experiment was repeated at least twice. The software SigmaPlot (version 12.0, Systat Software, Inc.) was used to calculate the XIP concentrations using the relative light unit (RLU) values.

### Genetic Transformation

Overnight cultures of all *S. mutans* strains were diluted 1:10 in fresh CDM and incubated in 5% CO_2_ at 37°C. The OD_600_ was followed until the cultures reached an absorbance value of 0.6, then 10% of glycerol was added and they were stored at −80°C. To perform the assay the stored cultures were diluted in fresh CDM 1:10 to an OD_600_ of 0.05 and incubated in air for 2 h at 37°C. The OD was measured and aliquots of 150 μL of the bacterial suspensions were transferred to 1.5 mL microcentrifuge tubes. The plasmid pVA838 (Table 1) was added to all tubes at a final concentration of 1 μg mL^–1^ and XIP was added in the experimental group at a final concentration of 1,000 nM. The samples were then incubated in air for an additional 4 h at 37°C, at which time the OD was measured again and the bacterial suspensions were serially diluted (up to 1 to 10^6^) in PBS. The suspensions were plated in duplicate on THB agar and THB agar supplemented with erythromycin 10 μg mL^–1^. The plates were incubated in 5% CO_2_ for 48 h at 37°C before counting the colony-forming units (CFU). The number of genetically transformed cells was divided per total CFU to obtain the values for transformation frequency. Three independent experiments were performed to evaluate transformation in CDM.

In order to evaluate genetic transformation in the non-responsive strains, an independent experiment extending the incubation time to 10 h before plating was performed. In addition, at 6 h an extra load of pVA838 was added, which increased the final concentration to 2 μg mL^–1^. For the remaining non-responsive strains, transformation was attempted using the 6.3 kb aRJ02 chromosomal PCR amplicon with a kanamycin marker, which is expected to have a higher sensitivity for detection of transformability (Table 1; Morrison et al., 2015; Salvadori et al., 2017).

### Synthetic Pheromones

The synthetic XIP (ComS_11__–__17_; GLDWWSL) (Mashburn-Warren et al., 2010; Khan et al., 2012) and the 18-CSP (ComC_26__–__43_; SGSLSTFFRLFNRSFTQA) (Petersen et al., 2006) were synthesized by GenScript (GenScript Biotech Corporation, Piscataway, NJ, United States), both with an estimated purity of 98% (Khan et al., 2012). The stock solutions of both peptides were stored in small aliquots at −20°C. The final concentration used in the genetic transformation assays for XIP was 1,000 nM. For FRET-assays to investigate protease activity, the 18-CSP was synthesized using methoxycoumarin-acetic-acidyl (MCA) on the N-terminal and Lys-Dinitrophenyl on the C-terminal (Dnp) (MCA-SGSLSTFFRLFNRSFTQA-Dnp; GenScript). On cleavage of the peptide by potential proteases, the Dnp quencher separates from the MCA fluorophore. The fluorophore is then activated giving an increase in fluorescence.

### FRET-18CSP Peptide Cleavage Reporter Assays

#### Growth in the Presence of FRET-18CSP

Pre-cultures of *S. mutans* were diluted in 2 mL CDM supplemented with 2% BSA to an OD_600_ of 0.04 to 0.05, and incubated at 37°C in air atmosphere to an OD_600_ of 0.06. The cultures were then distributed into the wells of a 96-well plate (115 μL in each well), and 5 μL of FRET-18CSP peptide was added (4 μM final concentration). The plate was incubated at 37°C in air, and fluorescence and optical density at 600 nm were measured at different time points during growth in a multi-detection microplate reader (Synergy, Cytation 3; excitation 325 nm, emission 392 nm).

#### Measurement of FRET-18CSP Cleavage in Culture Supernatants

Supernatants from *S. mutans* UA159 P*_*cipB*_*-luc reporter (SM059) and Δ*htrA* (SM165) were collected by centrifugation (6000 *g* for 10 min at 4°C) at different time points during growth, corresponding to early, mid- and late exponential phases. The supernatants were then distributed into the wells of a 96-well plate (115 μL in each well), and 5 μL of FRET-18CSP peptide was added (4 μM final concentration). The plate was incubated at 37°C in air atmosphere for 1 h, and fluorescence was measured in a multi-detection microplate reader (Synergy, Cytation 3; excitation 325 nm, emission 392 nm). Supernatants without FRET-18CSP were used for detection of background fluorescence, which were then subtracted from the values in the tested samples exposed to FRET-18CSP.

## Results

### Bioassay for CSP Detection in CDM

**FIGURE 2:**
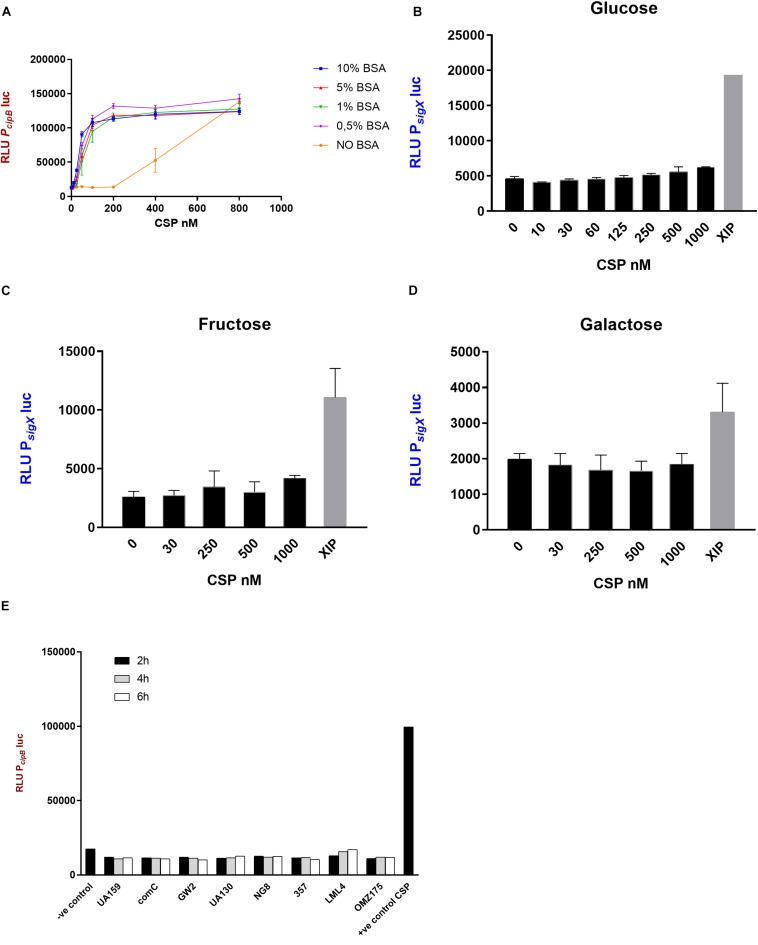
CSP activity in CDM. **(A)** The indicator strain SM059 (P*_*cipB*_*luc) was exposed to serial dilutions of synthetic CSP in the presence and absence of BSA. Different concentrations of BSA were tested. 10% BSA (blue), 5% BSA (red), 1% BSA (green), 0.5% BSA (pink), No BSA (orange). Bars correspond to standard errors of two to three independent experiments; RLU values were measured in a 96-well plate using a multi-detection microplate reader. **(B–D)** Activity of P*_*sigX*_*luc was determined after addition of various concentrations of CSP and 1 μM XIP in CDM supplemented with **(B)** glucose, **(C)** fructose and **(D)** galactose. Mean and standard deviation for two independent experiments. **(E)** Supernatants were collected from different *S. mutans* strains at 2 h (black bars), 4 h (gray bars) and 6 h (white bars) growth in CDM and examined for CSP activity using SM059 (P*_*cipB*_*luc) indicator strain. Medium alone was used as a negative control, and CSP (250 nM) was used as a positive control.

In this study we used a P*_*cipB*_*luc reporter for detection of extracellular CSP activity, in CDM as previously described (Khan et al., 2012), except that high sensitivity was only achieved in the presence of BSA (Figure 2A). BSA was chosen because it is known to enhance competence of *S. mutans* in rich media (Ahn et al., 2006), and in *S. pneumoniae*, BSA prevents CSP proteolysis (Cassone et al., 2012).

The potency of CSP in inducing the activity of the *cipB* promoter was increased by approximately 33-fold in CDM supplemented with BSA compared with CDM alone (Figure 2A). BSA concentrations from 0.5 to 10% gave similar results (Figure 2A).

### Failure of CSP to Induce *sigX* in CDM Was Independent of Carbohydrate Source

Since the induction of *cipB* was significantly enhanced in the presence of BSA, we decided to further examine the intriguing fact that CSP fails to stimulate *sigX* in CDM (Desai et al., 2012; Wenderska et al., 2012; Reck et al., 2015). Our hypothesis was that increased stimulation of the early response by CSP, as observed with BSA supplementation, would enable the activation of *sigX*, which is a late response in complex media. We used a similar bioassay as described above, but using the promoter of *sigX* linked to the luciferase gene instead. The results showed that CSP concentrations up to 1000 nM failed to induce the P*_*sigX*_*luc reporter in CDM supplemented with BSA and glucose (Figure 2B).

Since the carbohydrate source, at least in complex media, has a large influence on the induction of *sigX* expression by CSP (Moye et al., 2016), we also investigated *sigX* expression in CDM supplemented with fructose and galactose (Figures 2C,D). CSP still failed in demonstrating *sigX* induction. Thus, although CSP induces strong expression of *cipB* in CDM supplemented with BSA and different sources of carbohydrate, it fails to induce the late response characterized by the induction of *sigX* expression at concentrations as high as 1000 nM.

### Endogenous CSP Activity Was Not Detected in the Supernatants of *S. mutans*

Lack of endogenous CSP activity in the supernatants of *S. mutans* UA159 grown in CDM has been previously reported by our group (Khan et al., 2012). It is, however, unknown whether this is a conserved feature in the species. We investigated 6 other strains of *S. mutans*, and the *comC* deletion mutant SM004, for the CSP activity in their supernatants by growing them in CDM in the presence of BSA (Figure 2E). No *cipB*-inducing activity was detected in culture supernatants collected at early-, mid- or late- exponential phases of growth, thus indicating that the lack of extracellular CSP activity under such growth conditions may represent a conserved feature in *S. mutans*.

### Suppression of CSP Activity During Growth in CDM

**FIGURE 3:**
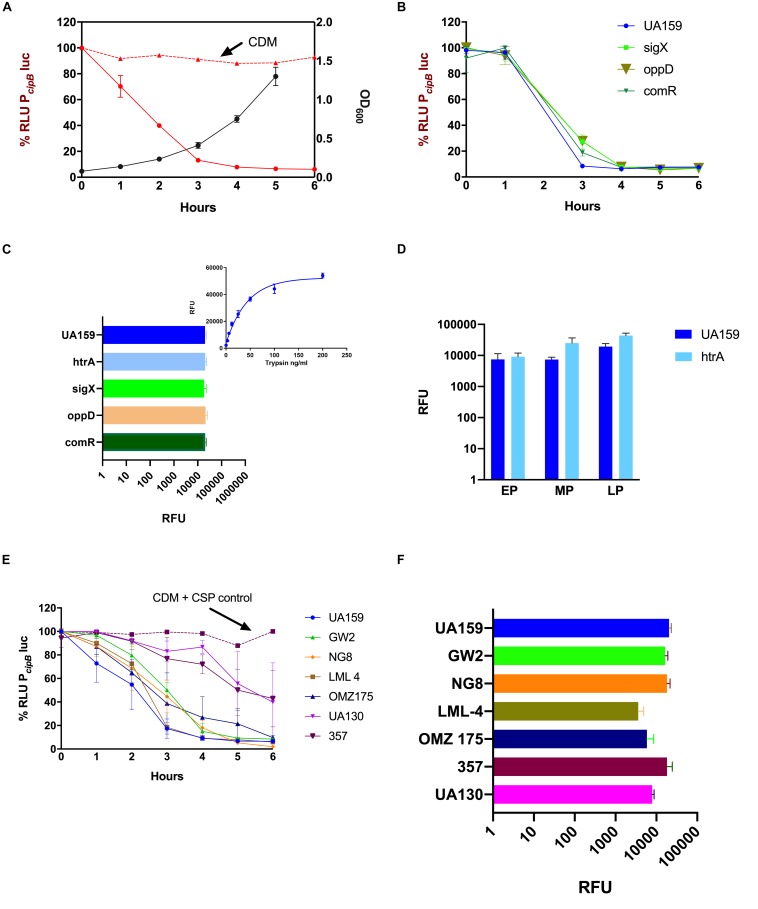
Suppression of CSP activity during growth in CDM. Frozen stock cultures at OD_600_ 0.6 were diluted 10× in CDM containing 10% BSA, and 250 nM synthetic CSP **(A,B,E)** or 4 μM FRET-CSP **(C,D,F)**. **(A,B,E)** The P*_*cipB*_*luc reporter was used to measure CSP concentration in the supernatants of different strains **(A)** *S. mutans* UA159 growth curve was measured at OD_600_ (black line) and indicates the time points when supernatants were collected. CSP activity in CDM alone (positive control) is represented by the red dashed line and in the supernatants of *S. mutans* UA159 by the red solid line. **(B)** Loss of CSP activity in the supernatants of UA159, Δ*sigX*, Δ*oppD* and Δ*comR.* **(C,F)** CSP cleavage measured at 240 min growth in the presence of the FRET-18CSP peptide, and recorded as relative fluorescence units (RFU) for panel **(C)** UA159, Δ*htrA*, Δ*sigX*, Δ*oppD*, and Δ*comR*, and **(F)** UA159, GW2, NG, LML-4, OMZ175, 357, and UA130. In panel **(C)**, right upper corner, a standard curve showing degradation of FRET-18CSP by trypsin is included as a reference. **(D)** FRET-18CSP proteolytic activity in supernatants of UA159 and the Δ*htrA* mutant collected at early (EP), mid- (MP) and late (LP) exponential phase of growth. RFU background values of the corresponding strains without FRET-CSP were subtracted. Error bars show standard error of mean from two to three independent experiments, with three parallels each.

Supernatants of *S. mutans* cultures exposed to 250 nM synthetic CSP were collected at different time points during growth in CDM. CSP activity in the supernatants of *S. mutans* UA159 was measured by using the *cipB* luciferase reporter described above. Within the first 3 h, CSP had almost completely disappeared from the culture supernatants (Figure 3A).

Due to the recognizable role of CSP in the induction of competence, it was crucial to determine if the loss of CSP activity was dependent on the development of competence. For this, we investigated the CSP activity in the culture supernatants of UA159 deletion mutants for *sigX*, *oppD*, and *comR* (Table 1). In all of them, a dramatic reduction in activity of exogenously added CSP was observed (Figure 3B).

To determine whether such an inhibitory effect on CSP activity could be due to proteolytic cleavage, we supplemented the CDM medium with a FRET-18CSP reporter peptide (Figure 3C). We found that all three isogenic mutants (*sigX*, *oppD*, and *comR*) grown under such conditions promoted increase in fluorescence activity, as measured after 240 min incubation at 37°C (Figure 3C), thus suggesting that the ability of *S. mutans* to degrade CSP does not require expression of key elements of the competence regulon. We also examined the effect of inactivating the *htrA* gene coding for the *S. mutans* HtrA protease, which in *S. pneumoniae* inactivates the CSP and cleaves misfolded proteins. No reduction in CSP degradation was observed for the *htrA* mutant (Figure 3C). CSP activity was indeed slightly higher for the culture supernatants of the *htrA* mutant at mid- and late exponential phases of growth (Figure 3D).

Finally, we tested whether suppression of CSP activity is extended to other *S. mutans* strains. In all the strains tested, reduction in CSP activity was observed, though at different levels (Figure 3E). When grown in the presence of the FRET-18CSP reporter peptide, an increase in fluorescence was observed for all strains (Figure 3F). We conclude that *S. mutans* suppresses CSP activity during growth in CDM, and that the mechanisms involved are active in the absence of *htrA*, *sigX, comR* or *oppD*, indicating the involvement of competence-regulated independent factors. The potential proteolytic activity leading to CSP inactivation seems to be conserved in *S. mutans*.

### XIP Activity Is Detected in the Culture Supernatants of Practically all *S. mutans* Strains Grown in CDM

**FIGURE 4:**
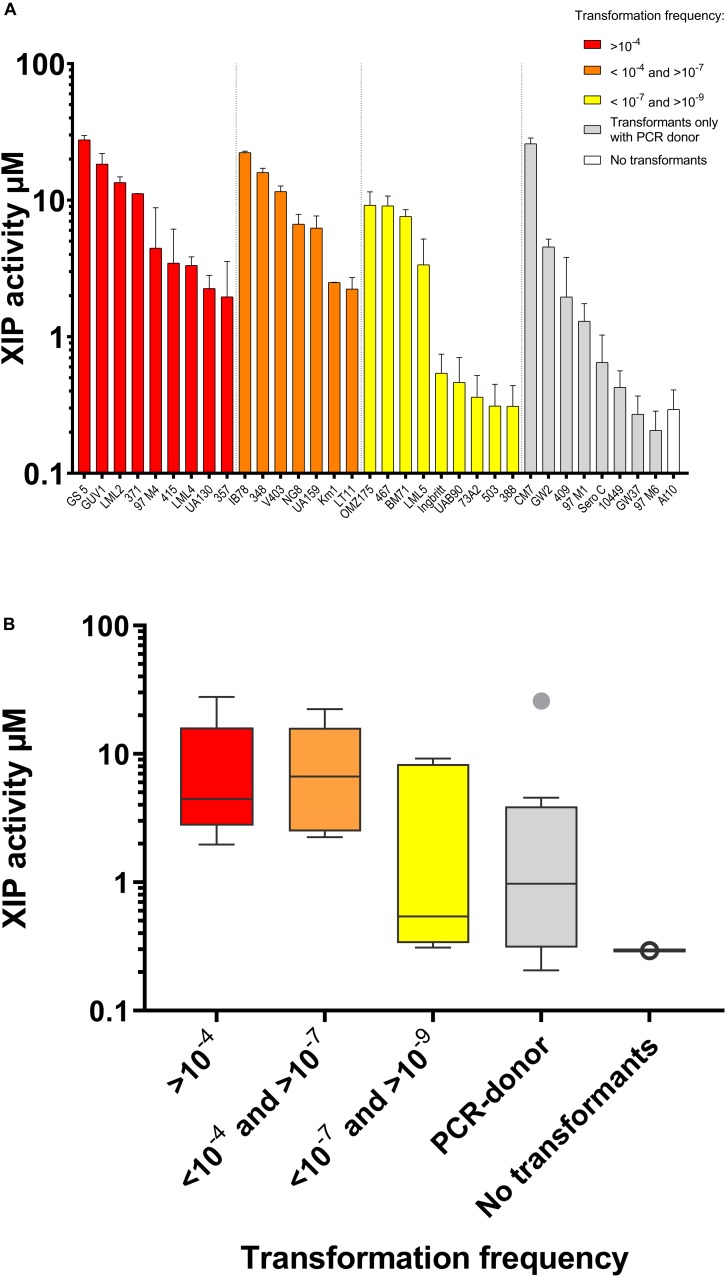
Conservation of XIP production and transformation in CDM. Extracellular XIP concentration was measured in the supernatants collected from *S. mutans* strains grown overnight at 37°C in air. **(A)** Columns show average of XIP-equivalent concentration from three independent experiments. Bars correspond to standard error. The indicator strain used was a Δ*comS* P*_*sigX*_*-luc reporter (SM091). Bars in different colors indicate transformation frequency in CDM supplemented with synthetic XIP by a plasmid donor (pVA838): (>10^–4^ in red, between 10^–4^ and 10^–7^ in orange, and between 10^–7^ and 10^–8^ in yellow). For those that were not transformed by the plasmid donor, transformation frequency with a 6.3 Kb PCR donor designed to be integrated into the chromosome by homologous recombination are shown (all frequencies below 10^–7^; gray). Only At10 yielded no transformants (white). **(B)** Whisker plots showing the average of XIP activity of the strains shown in panel **(A)**, grouped according to transformation frequency.

*S. mutans* UA159 produces and responds to exogenous XIP in CDM (Mashburn-Warren et al., 2010; Desai et al., 2012). We investigated whether XIP production is conserved in *S. mutans*. We used the P*_*sigX*_*luc Δ*comS* indicator (SM091) to estimate the XIP concentration in the supernatants of the different strains during growth. The minimum detection level was set at 10 nM, as determined by standard curve measurements using synthetic XIP. Our results showed the presence of extracellular XIP activity in all strains tested. The highest XIP concentrations in the supernatants of growing cultures ranged from approximately 200 to 26,000 nM (Figure 4A), with most showing XIP accumulation starting at the mid-exponential growth phase and reaching maximal values at early stationary phase (data not shown). We conclude that the growth conditions that support extracellular XIP pheromone accumulation by UA159 may also sustain XIP production by a majority of the strains.

### Thirty-Three Out of Thirty-Four Strains Were Naturally Transformable

Natural transformation without the addition of synthetic pheromone was detected in 23 of the 34 strains (data not shown). Because optimal levels of XIP may not be present at the time of competence under *in vitro* conditions, it was of interest to learn whether addition of synthetic XIP would result in higher transformation levels. Our results showed that synthetic XIP extended the range of transformed strains. Three of the strains with detectable XIP levels in the supernatants, including OMZ175, UAB90, and KM1 were transformed only upon addition of synthetic XIP. In the strains that were not transformed with the initial protocol, we increased the time during which they grew in the presence of synthetic XIP and donor DNA to 10 h, but even then no transformants were obtained (Murchison et al., 1986). We next used a more sensitive protocol based on the use of PCR large fragments as donor DNA (Morrison et al., 2015; Salvadori et al., 2017). With addition of the synthetic pheromone, 33 out of 34 strains were naturally transformable. Only strain At10 was not transformed, thus indicating that a majority of *S. mutans* are amenable to transformation. Overall, strains that exhibited lower transformation frequencies showed lower levels of XIP activity in their culture supernatants (Figure 4B).

### The *comS* Gene Was Conserved in the 34 *S. mutans* Strains

**FIGURE 5:**
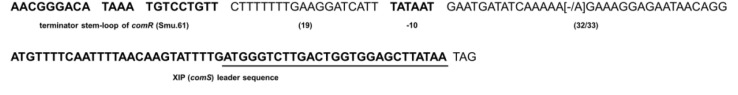
Conservation of *comS* promoter and *comS* sequences in *S. mutans* strains. The first sequence in bold shows the terminator stem-loop of *comR* (Smu.61) thought to function as part of the *comS* promoter. Downstream the stem-loop is a 19 bp sequence, followed by the putative –10 element of the *comS* promoter distant 32 to 33 bases from the *comS* gene sequence shown in bold. Underlined is the sequence corresponding to the XIP functional heptapeptide (ComS_11__–__17_ – GLDWWSL). Variable bases compared with *S. mutans* UA159 are in brackets.

The *S. mutans comS* gene encoding the XIP precursor is essential for competence development. Given the variability in transformation efficiency observed above, and that some strains were not amenable to transformation, we investigated whether the *comS* gene and its putative promoter sequences were conserved in the *S. mutans* strains included in the study. The results showed that *comS* was identical in all 34 strains (Figure 5). Highly conserved sequences were also found in the *comS* putative promoter. In this region the only difference in relation to UA159 was the presence or absence of an additional adenine between the −10 element and the *comS* translation initiation site. The additional adenine was present in 22 out of the 34 strains evaluated. In all the strains, *comS* was located 57–58 nt downstream of the tRNA transcriptional terminator of *comR* (SMU_61) and the putative −10 element was 32–33 nt upstream of the *comS* translation initiation site. While this study was being performed, new genomic sequences for 78 more strains of *S. mutans* were made available on public databases (Maruyama et al., 2009). In order to compare our results with the new genomic sequences we ran BLAST using the sequence shown in Figure 5 against the CoGe database^1^ (Lyons et al., 2011). The *comS* sequences in these strains were identical to the *S. mutans* UA159. In the *comS* promoter the sequences varied in only a single base pair at the same position as that observed in 22 of the strains sequenced in our study. Taken together the results indicate that the *comS* gene and promoter regions are highly conserved in the *S. mutans* strains analyzed, suggesting that variations in transformation levels and XIP production are not correlated with differences in this region.

## Discussion

In complex media, the CSP pheromone triggers the expression of bacteriocin-related genes, followed by a late response characterized by increased expression of the XIP-encoding gene *comS* and competence development (Kreth et al., 2005; Lemme et al., 2011; Khan et al., 2016). The mechanisms leading to activation of *comS* expression and competence remain unknown. It is also still unknown why CSP fails in stimulating competence in peptide-free defined medium. It has been suggested that *S. mutans* may perhaps produce a protease that, similar to the HtrA protease in *S. pneumoniae*, could inactivate the CSP (Desai et al., 2012). This possible explanation did not seem to match with the later finding that CSP is actually active in CDM, in that it can stimulate *cipB* and all other genes regulated by ComED (Reck et al., 2015). Our results show that *S. mutans* can indeed inactivate CSP by a mechanism that most probably involves proteolysis, given the results obtained with the FRET-18CSP reporter peptide. We confirmed previous results that CSP induces *cipB* (Reck et al., 2015) and found that a CSP concentration as low as 1 nM in the presence of BSA was sufficient to activate the early bacteriocin response. This is opposite to the CSP concentration to activate *sigX*, which requires values that exceed the maximum concentration used in this study (1000 nM) (data not shown). A higher threshold for *sigX* activation by CSP has also been observed in complex media (Son et al., 2012). A question that remains is why the threshold of CSP concentration to activate *sigX*, and therefore competence, is higher than for *cipB* activation, when CipB is thought to be the main link of CSP to *sigX* activation. One possibility is that at high concentrations CSP may activate or repress other two-component systems than ComED, which would then trigger a different link to competence. It is a well known phenomenon that although pheromones are usually highly specific to their cognate receptors, at high concentrations they may bind to other non-specific receptors (Hawver et al., 2016). However, several other mechanisms are possible, since the competence system is part of a signaling network of high complexity, affected by a variety of other systems not directly regulated by ComED, as recently reviewed (Kaspar and Walker, 2019). Irrespective of the mechanism, the higher threshold for CSP to induce competence indicates that CSP degradation may affect the competence response to this pheromone.

Inactivation of the *S. mutans* CSP by proteases produced by other streptococcal species has been known for more than 10 years (Wang and Kuramitsu, 2005). However, this is the first indication that *S. mutans* can also inhibit the activity of its own CSP, most probably via proteolytic activity. The genomes of *S. mutans* have more than 65 known or putative proteases, according to the MEROPS database^2^, but only HtrA has shown a role in autologous CSP cleavage. However, unlike for *S. pneumoniae* (Cassone et al., 2012), the HtrA protease in *S. mutans* was not required for CSP inactivation.

In *S. pneumoniae*, luciferase and *gfp* reporter strains for CSP activity have been successfully used to identify pneumococcal CSP pheromones in culture supernatants (Moreno-Gamez et al., 2017). Also in the seminal study that identified the *S. pneumoniae* competence factor, the CSP was isolated and purified from the supernatant (Havarstein et al., 1995). In *S. mutans* UA159 grown in complex medium, endogenous CSP has been identified by mass spectrometry in the supernatant fraction (Hossain and Biswas, 2012). Based on these findings, we hypothesized that if *S. mutans* produces CSP in CDM, we would probably be able to detect it in its culture supernatant. However, despite the high sensitivity of the protocol used in the present study, we could not detect any CSP-inducing activity in the supernatants of the different strains tested. Thus, if CSP is produced, it is either rapidly degraded outside of the cells or it remains associated with the cells.

In contrast to CSP, endogenous XIP activity was present in the supernatants of most strains. Moreover, activation of the XIP system in CDM enabled transformation of all but one of the 34 strains tested. The *comS* gene encoding the pre-processed form of XIP was indeed found in all the strains, which is in line with the results of a recent study reporting *comS* as part of the *S. mutans* core genome (Cornejo et al., 2013). In general, higher levels of XIP detection in the supernatants correlated with increased transformability. Of note, some strains that produced high concentrations of endogenous XIP needed to be stimulated by synthetic XIP to transform. This was, however, not surprising, given the fact that maximum concentrations were in most cases observed only close to the stationary phase, which is a period during which competence under laboratory conditions is already shut off. For some of the strains that produced low levels of XIP, transformation was only detected by using a large PCR amplicon as DNA donor, which is known to result in increased recovery of transformants (Morrison et al., 2015; Junges et al., 2017).

Taken together, our results indicate that the XIP system is conserved in *S. mutans*, and that all *S. mutans* strains may belong to a single pherotype. Moreover, the conserved ability of *S. mutans* to cleave CSP under conditions that favor accumulation of XIP may provide an adaptive advantage to *S. mutans*, by allowing them to fine-tune competence and bacteriocin responses in response to environmental changes. While the knowledge on pheromone signaling for any species is mostly based on models derived from limited selected strains, understanding how the system works in different strains of a species is of high relevance for the development of strategies aiming at interfering with their communication systems.

## Data Availability

All datasets generated for this study are included in the manuscript and or supplementary files.

## Author Contributions

All authors conceptualized the manuscript, drafted and critically revised the manuscript and approved the final version of the manuscript for publication.

## Conflict of Interest Statement

The authors declare that the research was conducted in the absence of any commercial or financial relationships that could be construed as a potential conflict of interest.

## Footnotes

1 http://genomevolution.org/CoGe/CoGeBlast.pl

2 https://www.ebi.ac.uk/merops/index.shtml
